# The effect of Shexiang Tongxin Dropping Pills on coronary microvascular dysfunction (CMVD) among those with a mental disorder and non-obstructive coronary artery disease based on stress cardiac magnetic resonance images: A study protocol

**DOI:** 10.1097/MD.0000000000020099

**Published:** 2020-05-22

**Authors:** Jinfan Tian, Lijun Zhang, Xueyao Yang, Huijuan Zuo, Xin Zhao, Jingwen Yong, Yi He, Xiantao Song

**Affiliations:** a Department of Cardiology; b Department of Radiology; c Department of Community Health Research, Beijing Anzhen Hospital, Capital Medical University; d Department of Radiology, Beijing Friendship Hospital, Capital Medical University, Beijing, China.

**Keywords:** coronary microvascular dysfunction, non-obstructive coronary artery disease, Shexiang Tongxin Dropping Pills, stress cardiac magnetic resonance, study protocol

## Abstract

**Introduction:**

Coronary microvascular dysfunction (CMVD), highly prevalent among patients with a mental disorder (anxiety or depression), is closely related to adverse cardiac events, including hospitalization, sudden cardiac death, and myocardial infarction. Shexiang Tongxin Dropping Pills (STDP), a traditional Chinese medicine, exerts endothelial protective function by anti-inflammation, anti-oxidative stress, and promoting blood circulation. STDP protects against CMVD in previous fundamental studies. The present trial is aiming at evaluating the effect of STDP on CMVD among depressed or anxious patients with non-obstructive coronary artery disease (NOCAD).

**Methods and analysis:**

Seventy-two depressed or anxious patients diagnosed with NOCAD combined with CMVD utilizing coronary artery angiography and stress cardiac magnetic resonance (CMR) will be recruited in the present study. These patients will be randomized into two groups, namely, Nicorandil group (Nicorandil combined with routine medicine), and STDP groups (STDP combined with routine medicine). The change of CMVD status by assessing absolute myocardial blood flow and myocardial reperfusion using stress CMR 3-month after discharge is defined as the primary endpoint. Major adverse cardiac events (MACEs), quality of life (QOL), and metal disorder improvement are defined as the secondary endpoints. Seattle angina questionnaire (SAQ) which is used to assess angina pectoris and QOL will be recorded at 1-, 3-, 6-, 9-, 12-month of follow-up. Seven-item Generalized Anxiety Disorder Scale (GAD-7) and 9-item depression module from the Patient Health Questionnaire (PHQ9) which utilized to evaluate anxiety and depression, respectively, will be recorded at 1-, 3-, 6-, 9-, 12-month of follow-up. This study will first evaluate the efficacy of STDP on CMVD among patients with a mental disorder and NOCAD, and discuss the potential mechanisms, providing therapeutic evidence for the STDP for these patients.

## 1 Introduction

A large proportion of patients with angina pectoris are found non-obstructive coronary artery disease (NOCAD) when undergoing coronary angiography. Currently, CMVD has aroused great attention among cardiologists after the officially published of the *Chinese Expert Consensus on Diagnosis and Treatment of Coronary Microvascular Disease* written by Dr Zhang Yun. Coronary microvascular dysfunction (CMVD) is highly prevalent among patients with chest pain and tightness complaints. Among those with symptoms of myocardial ischemia and NOCAD verified by coronary angiography, the incidence of CMVD ∼45% to 60%.^[1,2]^ A study published in the *circulation journal* in 2014 reported that among 405 males and 813 females without a history of coronary heart disease, the incidence of CMVD was 51% and 54%, respectively, based on the coronary flow reserve (CFR) <2 measured by PET.^[3]^ CFR < 2 measured by PET was an independent predictor for adverse cardiovascular events. The patients with CMVD are at great risk of adverse cardiovascular events, including re-hospitalization due to heart failure, sudden cardiac death, myocardial infarction, etc. Nowadays, a mental disorders including anxiety and depression have been found associated with adverse cardiovascular events. However, still, a large proportion of the patients with a mental disorder have no significant stenosis in the epicardial artery. Owing to the association between a mental disorder and impaired endothelium-dependent vasodilatation, endothelium-independent vasodilatation, and hyperactive inflammation or oxidative stress,^[4]^ we hypothesized that patients with anxiety or depression are more likely to have CMVD compared to those without a mental disorder, and CMVD is one of the factors for a mental disorder contributing to adverse cardiovascular events. Therefore, preventing CMVD among those with a mental disorder and NOCAD is of great significance.

The insight into pathogenesis promotes the CMVD diagnosis and therapy. Either endothelium-dependent or endothelium-independent vasodilatation disorder contributes to CMVD. Impaired endothelium-dependent NO is associated with endothelium-dependent vasodilatation; impaired opening of ATP-dependent K+ channels contributes to endothelium-independent vasodilatation.^[5]^ Additionally, inflammation and oxidative stress are established as the mechanisms for CMVD.

The diagnosis of CMVD is composed of noninvasive and invasive manner. The invasive Index of Microcirculatory Resistance (iMR) based on coronary angiography is associated with diagnosis and prognosis of CMVD. In patients with stable angina and non-obstructive coronary artery disease, CMVD can be defined as iMR threshold ≥25 U, and elevated iMR is associated with major adverse cardiac events. Noninvasive diagnosis depends on the measurement of coronary vasomotor function by evaluation regional and global myocardial blood flow at rest and during stress. Cardiac magnetic resonance (CMR) is accurate in quantification of regional and global myocardial perfusion using semiquantitative myocardial perfusion reserve index (MPRI) or quantitative myocardial perfusion reserve (MPR). CMR imaging protocol is compromised by of a rest and vasodilator-stress first-pass myocardial perfusion testing, following the injection of a gadolinium-based contrast agent. T1 mapping, a gadolinium-free stress CMR approach has also been used for diagnosis of CMVD.^[6]^ When impaired MPR is detected by invasive testing or by noninvasive quantification of MBF in NOCAD, it is assumed that the angina is attributable to CMVD.

Adenosine stress CMR is applied in this study, owing to the fact that it is a feasible noninvasive approach for assessing microvascular function with its high accuracy, spatial resolution, and radiation-free. According to Benjamin Zorach et al, in patients with risk factors for CMVD, both MPR (2.21 [1.95, 2.69] vs 2.93 [2.76, 3.19], *P* < .001) and stress myocardial perfusion (2.65 ± 0.62 mL/min/g, vs. 3.17 ± 0.49 mL/min/g, *P* < .002) were reduced as compared to controls. Consequently, they concluded that stress myocardial perfusion and MPR are reduced in patients with risk factors for CMVD with NOCAD as compared to healthy controls.^[7]^ CMR is well correlated with invasive approach in diagnosis of myocardial dysfunction. According to Alexander Liu et al.^[8]^ in patients with non-obstructive coronary artery disease, myocardium with IMR < 25 U had normal MPRI (1.9 ± 0.4 vs controls 2.0 ± 0.3; *P* = .49); myocardium with IMR ≥ 25 U had significantly impaired MPRI, demonstrating that CMR can objectively and noninvasively assess microvascular angina in patients with non-obstructive coronary artery disease.

Currently, there is lack of effective therapeutic approach targeting CMVD. The value of available therapeutic trials is limited by non-selected patients due to the lack of standardized diagnosis, and study designs with small sample size. The available therapeutics include risk factor control, anti-anginal therapies (nitrates, β-blockers, and calcium channel blockers), and anti-ischemic drugs (nicorandil, ivabradine, and aminophylline). Nicorandil contributes to vasodilatation of the major epicardial artery and microvascular by mechanically opening ATP-sensitive potassium channel. An early study suggested that intravenous administration of nicorandil reduced microvascular dysfunction among patients with stable angina undergoing percutaneous coronary intervention (PCI) for left anterior descending artery lesion.^[9]^ Another triple-blind and randomized clinical trial revealed that nicorandil effectively improved tissue perfusion after PCI and reduced damages caused by angioplasty among patients with stable angina pectoris.^[10]^ Although nicorandil is first recommended in CMVD treatment, the wide use is limited by its side effects such as headache and gastroenteric symptom. Consequently, an effective and well tolerant drug is urgent for CMVD therapy, especially for these patients with depression or anxiety.

STDP, a traditional Chinese formula, consists of a combination of Moschus, Radix et Rhizoma Ginseng, Calculus Bovis, bear gall, Venenum Bufonis, borneol, and salvia miltiorrhiza. Thirteen active compounds (ginsenoside Rg1, ginsenoside Rk3, cinobufagin, arenobufagin, bufalin, resibufogenin, tanshinone IIA, taurine, tauroursodeoxycholic acid, taurocholic acid, cholic acid, deoxycholic acid, and chenodeoxycholic acid) are identified in STDP using ultra-performance liquid chromatography coupled with triple-quadrupole tandem mass spectrometry in multiple-reaction monitoring mode.^[11]^ Currently, STDP is a prescription medicine approved by traditional China Food and Drug Administration (CFDA) (approval NO. Z20080018). Previous animal studies showed that STDP was effective in attenuated myocardial ischemic injury.^[12,13]^ The clinical STDP revealed its efficiency in ischemic chest pain,^[14,15]^ the mechanisms involving anti-inflammation and endothelial function protection.^[16]^ Furthermore, due to its good tolerance and multi-effect of STDP, it is a promising therapeutic approach for CMVD treatment among patients with mental disease and NOCAD. Hence, we aimed at evaluating the efficiency of STDP in patients with CMVD and a mental disorder but without obstructive coronary artery disease. Furthermore, the potential mechanism will be discussed in the study.

## 2 Methods and analysis

### 2.1 Study design

**Figure 1:**
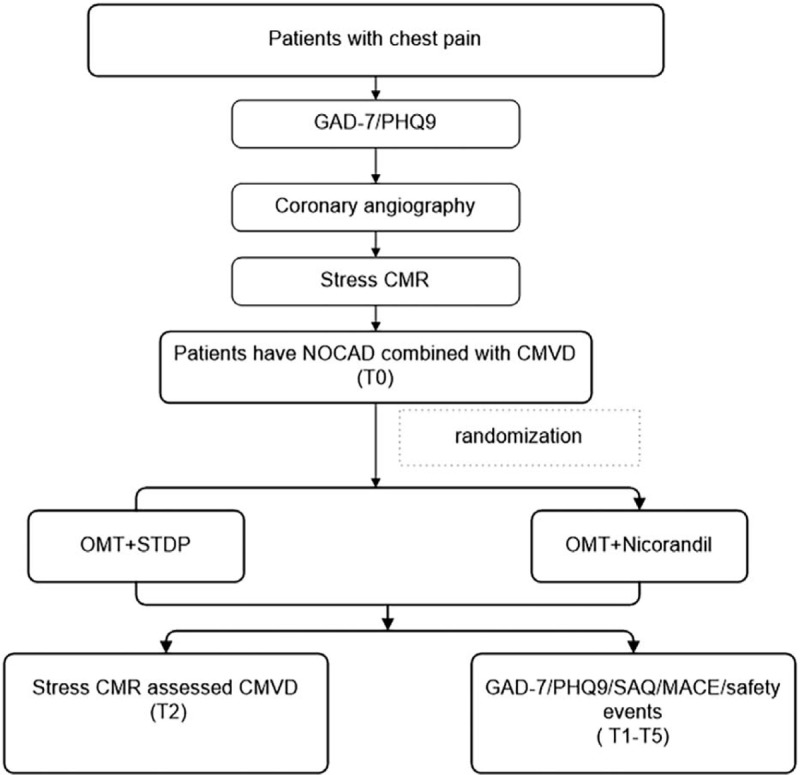
Study protocol. Patients who have chest pain and a mental disorder, combined with significant epicardial coronary artery stenosis less than 50% assessed by coronary angiography will be primarily screened. CMR at rest and adenosine stress is utilized to further screen the eligible participants. The eligible participants will be randomized into two groups, namely the OMT + STDP group (n = 36) and the OMT+ nicroandil group (n = 36), following the signature of the informed consent. The primary endpoints are coronary microvascular index (MBF, MPR, and MPRI) changes determined by CMR at T2. The secondary endpoints are changes of SAQ, GAD-7, PHQ-9, SAQ, and MACE events at follow-up (T1–T5). The safety events will be recorded at T1–T5. CMR = cardiac magnetic resonance image, GAD-7 = 7-item Generalized Anxiety Disorder Scale, NOCAD = non-obstructive coronary artery disease, OMT = optimal medicine treatment (aspirin, statin, nitrates, β-blockers, angiotensin-converting enzyme, angiotensin II antagonist, calcium channel antagonist) are administered according to the individual's basic conditions), PHQ9 = 9-item depression module from the Patient Health Questionnaire, STDP = Shexiang Tongxin Dropping Pills. T1: 1 month after medication; T2: 3 months after medication; T3: 6 months after medication; T4: 9 months after medication; T5: 12 months after medication.

This study will be performed using prospectively, single-center, and randomized design. All the study protocol was registered in the Chinese clinical Trial Registry (ChiCTR1900025810). Seventy-two patients with CMVD and NOCAD, who meet the inclusion criteria will be randomized into two groups, namely, treatment group (n = 36) and control group (n = 36). The randomized digital form and interactive voice response mode will be applied to implement the randomization procedure. The randomization sequence will be produced and managed by an independent staff. Clinical measurement will be performed at primary screening period and at baseline (before randomization, T0). STDP will be administered for 12 months. The outcomes and safety index will be measured at months 1 (T1), 3 (T2), 6 (T3), 9 (T4), and 12 (T5) after medication. Figure 1 showed the study protocol. The protocol has been approved by ethics committee of Beijing Anzhen Hospital, China (approval NO. KS2019014).

### 2.2 Participants

A total of 72 patients with a mental disorder and NOCAD hospitalized in Beijing Anzhen Hospital and diagnosed with CMVD will be consecutively recruited in this study from October 2019 to October 2021. An independent staff obtained the informed consent before the program.

#### 2.2.1 Inclusive criteria

1. Those with chest pain (including stable angina pectoris and unstable angina pectoris);
2. Those aged between 18 and 70 (contain patients aged 18 and 70);
3. Those with left ventricular eject fraction more than 50% assessed by transthoracic echocardiography;
4. Those with anxiety (7-item Generalized Anxiety Disorder Scale [GAD-7] ≥5) and/or depression (9-item depression module from the Patient Health Questionnaire [PHQ9] ≥ 5);
5. Those with no obvious stenosis (>50%) in the major epicardial artery, or with no more than 50% stenosis in the original PCI artery;
6. Those fully understand the research requirements and intervention procedures, and signed informed consent before the program;
7. Those comply with all examination and follow-up protocol;

#### 2.2.2 Exclusive criteria

1. Those with new-onset myocardial infarction within the latest 3-month;
2. Patients who had CABG;
3. Patients who are allergic or contraindicate to antiplatelet or anticoagulation drug, contrast, nicorandil or STDP;
4. Those with severe abnormal hematopoietic system, such as platelet counts <100∗109/L or >700∗109/L and white blood cell counts <3∗109/L;
5. Those with active bleeding or bleeding tendency;
6. Those with severe kidney dysfunction (GFR < 60 mL/min^●^1.73 m^2^);
7. Those with severe hepatic dysfunction (ALT or AST ≥ 3-fold of the upper limit of the normal reference);
8. Those with severe heart failure (NYHA classification ≥ III);
9. Those suffering from tumor and other disease with life expectancy less than 12 months;
10. Women who are pregnant or plan to be pregnant;
11. Those with acute infectious diseases and immune disorders;
12. Those unable to communicate due to cognitive impairment, auditory and visual impairment;
13. Patients who are participating in or plan to participate in another clinical trial;
14. Those with claustrophobia.

### 2.3 Withdraw and discontinuation

Patients withdraw because of complications, adverse safety events or inability to observe the medication protocol will be taken into analysis in Full analysis set (FAS) instead of Per Protocol Set (PPS). The reason for withdrawing and the final medication status will be recorded. The primary endpoint data of the patient who has MACE, non-cardiac death, or other disease that affect expect life span within 1-year, will be considered censored. The Baseline measurements of patients who have MACE will be analyzed.

### 2.4 Intervention

All the patients will be given standard dual-antiplatelet therapy (aspirin combined with a P2Y12 receptor antagonist) before coronary angiography. The patients met the inclusion and none of the exclusion criteria will be randomized into two groups:

1. treatment group, STDP (2 pills/time, 3 times/day) + other drug;
2. control group, nicorandil +other drug.

Other drug treatments (aspirin, statin, nitrates, β-blockers, angiotensin-converting enzyme, angiotensin II antagonist, and calcium channel antagonist) are administered according to the individual's basic conditions.

### 2.5 Endpoints

#### 2.5.1 Primary endpoint

CMVD status (myocardial blood flow [MBF], MPR, and MPRI) changes of each group at T2 (Table 1); MBF, MPR, and MPRI differences between treatment group and control group.

**Table 1:**
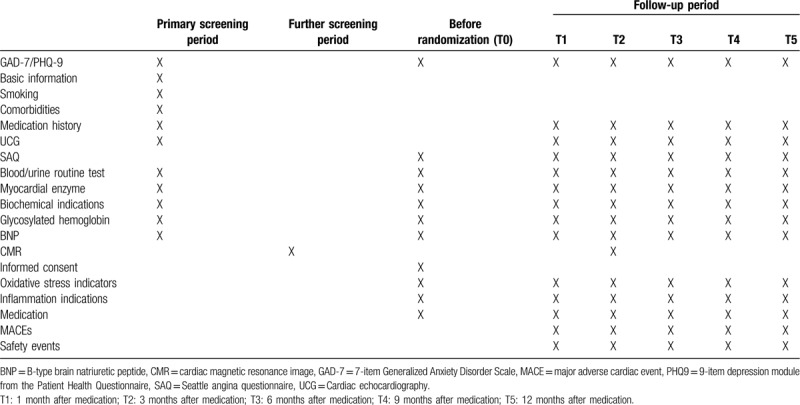
Study procedural and visual schedule.

#### 2.5.2 Secondary endpoints

1. The difference of MACEs (non-fatal myocardial infarction, vascularization, cardiac death, heart failure, cardiac rehospitalization, and stroke) between treatment group and control group (T1–T5);
2. A mental disorder changes of each group (assessed by GAD-7 and PHQ9) at T1–T5 (Table 1); GAD-7 and PHQ9 differences between treatment group and control group;
3. Quality of life (QOL) and angina pectoris occurrence changes (both assessed by Seattle angina questionnaire, SAQ) of each group at T1–T5 (Table 1); SAQ differences between treatment group and control group;
4. Inflammation (hsCRP) and oxidative stress index (SOD and MDA) changes at T1–T5 (Table 1); hsCRP, SOD, and MDA differences between treatment group and control group;
5. Cardiac function changes at T1–T5 (Table 1); cardiac function differences between treatment group and control group.

#### 2.5.3 Safety endpoints

Liver and renal function impairment (assessed by ALT, AST, Cr, and BUN), and gastrointestinal reaction will be recorded at T0 to T5 during following-up (Table 1).

### 2.6 Assessment of anxiety and depression

GAD-7 will be used for diagnosing anxiety. GAD-7 scores range from 0 to 21, with scores of ≥5, ≥10, and ≥15 represent mild, moderate, and severe anxiety symptom levels, respectively.^[17]^ PHQ9 will be used for screening and assessing the severity of depression. A score of 5 to 9 indicates mild depression; 10 to 14 indicates moderate depression; a score 15 to 19 indicates moderate-severe depression; a score 20 to 27 indicates severe depression.

### 2.7 CMR protocol

The CMR was performed on a 3.0 T scanner according to the established techniques,^[7]^ including cine, adenosine stress and rest perfusion, and late gadolinium enhancement (LGE) imaging. The absolute quantification of MBF at rest and adenosine stress will be measured; MRP will be quantitatively calculated by the ratio of stress MBF to the resting MBF; MPRI will be obtained semi-quantitatively by the ratio of myocardial signal intensity upslopes, and it will be normalized to the arterial input function. According to Taqueti VR et al., MPRI < 2 is defined as CMVD.^[18]^

### 2.8 Study procedures and visual schedule

Patients who have chest pain and a mental disorder, combined with significant epicardial coronary artery stenosis <50% assessed by coronary angiography will be primarily screened. Cardiac echocardiography (UCG) is performed in the primary screening period. CMR at rest and adenosine stress is utilized to further screen the eligible participants. The eligible participants will be randomized into two groups following the signature of the informed consent. The basic information (age, gender, blood pressure, and body mass index), smoking, commodities (diabetes, hypertension, and PCI history) will be recorded at primary screening period. The medication will be recorded at primary screening period, during hospitalization, at discharge and at follow-up. CMR index (MBF, MPR, and MPRI) will be recorded at primarily screening period and at T2. Blood and urine routine test, myocardial enzyme (CK, CKMB, cTNI, and LDH), biochemical indications (GLU, TC, TG, ALT, AST, creatinine, and BUN), glycosylated hemoglobin, and BNP will be recorded at primarily screening period and at T0 to T5. Inflammation indications (hsCRP), oxidative stress index (SOD and MDA), SAQ, GAD-7, PHQ-9 will be recorded at T0 to T5. UCG index is determined at primary screening period and at T1 to T5. MACEs and adverse events will be recorded at follow-up (T1–T5).

### 2.9 Sample size calculation

PASS 15.0 is utilized to calculate sample size. For the main purpose of this trial is to investigate CMVD status change. Stress myocardial perfusion is taken into consideration for the calculation of sample size. According to previous study showing that 0.5 mean difference between CMVD patients and controls with respect to stress myocardial perfusion, the non-inferiority margin is set at the level of 0.3. And a value of 0.7 is defined as overall standard deviation (σ). With a type I error rate of α = 0.025 and a power of 90% (1−β), each group should be composed of 30 patients using a one-tailed test. Considering potential loss and withdraw (20%), 36 patients are needed for each group. Therefore, 72 patients are needed to be recruited in the study.

### 2.10 Data entry, monitor, and quality control

The case report form (CRF) will be finished by independent investigators. All the data will be documented in the database by two independent investigators. A special inspector is responsible for tracking, monitoring and correcting any errors to keep the electronic database consistency with the source documents. Database will be inactive after cross-validation, followed by the statistical analysis. The staff that performed analysis was blinded to the intervention.

### 2.11 Endpoint adjudication committee

An independent committee will be available to adjudicate and report the primary endpoint, secondary endpoint and safety endpoint. The committee has the authority to terminate the trial if any safety endpoint occurs.

### 2.12 Statistical analysis

In the FAS, all randomized patients will be kept in the treatment group to which they were originally randomized. Additionally, patients without protocol deviation will be included in the PPS. Means ± standard deviation is used to express continuous variables. Student's *t-*test will be used to compare two independent samples when the variables are normally distributed. The Mann–Whitney *U* test will be used to compare non-normally distributed data. Frequencies and percentages will be used to express categorical variables, and the chi-square test will be used to compare the data. The difference risk of MACE between the two groups will be adjusted for other factors such as age and comorbidities (diabetes mellitus, and hypertension). A 95% confidence interval will be used. A two-side *P* < .05 will be considered statistical significance.

## 3 Discussion

As aforementioned, CMVD is prevalent among the patients without obstructive coronary artery disease, associated with an adverse cardiac event and poor prognosis. It has also been established that depression contributes to CMVD and chest pain due to hyperactive inflammation and impairment of NO-dependent vascular relaxation.^[4]^ Up to now, there is a lack of evidence supporting the efficiency treatment on CMVD among patients with NOCAD especially those combined with mental disorders. The active compounds in STDP exert anti-inflammation, anti-oxidative stress, and blood circulation modulation effects. Furthermore, STDP has a beneficial effect on “Qi” according to the traditional Chinese Medicine theory. This study first evaluates the effect of STDP on CMVD among patients with NOCAD and a mental disorder. Endothelial function indicators, anxiety scale, and depression scale will be utilized to explore the potential mechanisms for STDP on CMVD in the selected patients. We hypothesized that STDP could protect against CMVD among the recruited patients by improving a mental disorder or by protecting endothelial function. The previous study by Wang et al.^[19]^ has made an attempt to investigate the effect of STDP on slow coronary flow which is associated with coronary microvascular dysfunction and endothelial dysfunction. The finding revealed that corrected thrombolysis in myocardial infarction frame count values of vessels with slow flow was significantly decreased after sublingual administration of STDP treatment. Zhang et al.^[20]^ revealed that STDP restored the lipopolysaccharides-impaired microcirculation flow velocity in myocardial infarct mice, and the mechanisms involved the increased expression of endothelial nitric oxide synthase (eNOS) and endogenous NO by STDP treatment. Based on these evidence, STDP is promising in treating CMVD among those with NOCAD.

## 4 Conclusion

To the best of our knowledge, this is the first study that evaluates the efficacy of STDP on CMVD among patients with a mental disorder and NOCAD, and discusses the potential mechanisms, providing therapeutic evidence for the useful of STDP for these patients. However, there are several limitations in our study. First, CMVD could be affected by other factors such as other new-onset disease and motions during follow-up, which limit the evaluation for real effect of STDP on CMVD. Second, 1-year of follow-up may be too short to observe the difference of MACE between the two groups. Hence, a further study with a longer period may be needed to evaluate the effect of STDP on MACEs among these patients. Third, endothelial protection function could be derived from the mental disorder improvement. Hence, in some instances, we are not able to discrete the protection effect of endothelium from a mental disorder improvement. Finally, the mental disorder improvement or endothelial protection maybe not fully explain all the effectiveness of STDP on CMVD.

## Acknowledgments

The authors highly thank for Wenyi Zhang and Haoran Xing who helped to recruit patients. We gratefully acknowledge Xueyao Yang, Bing Yu Gao for the patients’ follow-up, as well as Xin Zhao and Nan Nan for the data collection.

## Author contributions

**Conceptualization:** Xiantao Song

**Date management:** Jinfan Tian and Lijun Zhang

**Endpoint adjudication:** Xiantao Song and Yi He

**Investigation:** Xueyao Yang, Xin Zhao and Jingwen Yong

**Supervision:** Huijuan Zuo
